# Report of Deaths in the City of Buffalo, for the Months of January, February and March, 1864

**Published:** 1864-05

**Authors:** Sandford Eastman

**Affiliations:** Health Physician


*Report of Deaths in the City of Buffalo, for the Months of January, February and March,* 1864. By Sandford Eastman, M. D., *Health Physician.*

| | January. | February. | March. | Total. |
|---|---|---|---|---|
| Whole number of deaths from disease in | 125 | 117 | 142 | 384 |
| In addition to these the following still-born were reported | 3 | .... | 1 | 4 |
| Total | 128 | 117 | 143 | 388 |
| **LOCALITY.** | | | | |
| City at large | 116 | 99 | 124 | 339 |
| Sisters' Hospital | 6 | 6 | 5 | 17 |
| Buffalo General Hospital | 2 | 1 | 5 | 8 |
| Catholic Foundling Asylum | .... | 4 | 2 | 6 |
| Erie County Alms House | 3 | 5 | 5 | 13 |
| Erie County Penitentiary | 1 | .... | .... | 1 |
| Fort Porter | .... | 2 | 1 | 3 |
| Providence Insane Asylum | .... | .... | 1 | 1 |
| Total | 128 | 117 | 143 | 388 |
| **BY WHOM CERTIFIED.** | | | | |
| By regular Physicians at Public Institutions | 11 | 16 | 19 | 46 |
| By regular Physicians in city at large | 64 | 62 | 80 | 206 |
| By irregular Practitioners | 31 | 15 | 15 | 61 |
| By Coroner | 6 | 8 | 8 | 22 |
| By Undertakers | 16 | 16 | 21 | 53 |
| Total | 128 | 117 | 143 | 388 |

CAUSES OF DEATH, IN THE THREE MONTHS.

Accident, 7; do. by burn, 3; do. by drowning, 4; Albuminuria, 2; Apoplexy, cerebral, 7; Astnma, 1; Brain, congestion of 3; do. softening of 1; Bronchitis, 3; Cancer of stomach, 2; do. of womb, 1; Cholera infantum 1; Chorea, 1; Cirrhosis of liver, 2; Consumption, 54; Convulsions, 26; Croup, 12: do. diphtheritic, 4; Debility, 9; Delirium tremens, 6; Dentition, 1; Diarrhœa, 8; Disease of the brain, 1; do. heart, 6; do. liver, 1; do. lungs, 1; do. stomach, 1; Diphtheria, 11; Dropsy, general, 4; do. abdominal, 2: do. brain, 3; do. heart, 1; do. ovarian, 1; Dysentery, 1; Epilepsy, 3; Erysipelas, 5; Fever, 2; do. puerperal, 8; do. puerperal convulsions, 1; do. scarlet, 13; do. typhoid, 8; do. typhus, 3; Gangrene, 1; Gun-shot wound, 2; Hemorrhoids, 1; Hemorrhage from lungs, 1; do. uterus, 1; Inflammation knee-joint, 1; do. bowels, 7; do. brain, 2; do. brain and meninges, 11; do. lungs, 35; do. lungs. typhoid, 9; do. lungs and pleura, 3; do. peritoneum, 1; do. stomach, 2; do. womb, 3; do. appendix vermiformis, 2; Insanity, 3; Intemperance, 3; Intussusception, 1; Injury of spine, 1; Marasmus, 3; Measles, 3; Murder, (strangulation,) 1; Ovarian tumor, 1; Old age, 16; Paralysis, 3; Premature birth, 2; Pemphigus, 1; Pyæmia, 4; Rheumatism, 4; Shock of operation, 1; Scrofula, 2; Spina bifida, 1; Suicide, 1; Suffocation, 5; Tetanus, 1; Trachitis, 1; Unknown, 9; Wound in throat, 1.

The following shows the number of deaths in the City in each month of the present year; the number in 1863, and the average of each month for the five years, 1859 to 1863, inclusive:

| *Month.* | 1864. | 1863. | 5 *years' average.* |
|---|---|---|---|
| January, | 128 | 115 | 129 |
| February, | 117 | 94 | 117 |
| March, | 143 | 104 | 131 |

The number of deaths in the first three months of the present year is 75 *more* than in the corresponding period of last year; and 11 *more* than the average for five years.

SANDFORD EASTMAN, M. D.,
Health Physician.

